# Bergenin ameliorates airway inflammation and remodeling in asthma by activating SIRT1 in macrophages to regulate the NF-κB pathway

**DOI:** 10.3389/fphar.2022.994878

**Published:** 2022-10-13

**Authors:** Dan Huang, Chaoqun Sun, Min Chen, Shuyou Bai, Xuanna Zhao, Weiming Wang, Kang Geng, Wenbo Huang, Tingting Zhao, Bin Wu, Guilin Zhang, Dong Wu, Youhua Xu

**Affiliations:** ^1^ State Key Laboratory of Quality Research in Chinese Medicine, Macau University of Science and Technology, Taipa, China; ^2^ Department of Respiratory and Critical Care Medicine, Affiliated Hospital of Guangdong Medical University, Zhanjiang, China; ^3^ Guangdong Keguanda Pharmaceutical Technology Co Ltd, Guangzhou, China

**Keywords:** bergenin, SIRT1, NF-κB, asthma, macrophage

## Abstract

Airway inflammation and remodeling are critical pathological changes in asthma, and macrophage activation plays a vital role in this process. Sirtuin 1 (SIRT1) reduces airway inflammation by affecting macrophages in asthma. This study aimed to investigate the potential benefit and underlying mechanism of the SIRT1 agonist bergenin as a treatment for asthma. We performed *in vivo* and *in vitro* experiments by establishing a *Sirt1* ^*fl/fl*^ *-LysMcre* mouse asthma model and using the alveolar macrophage-like cell line MH-S, respectively. Our results show that *Sirt1* ^*fl/fl*^ *-LysMcre* asthmatic mice exhibited more severe airway inflammation and airway remodeling than wild-type mice. As an activator of SIRT1, bergenin attenuated asthmatic airway pathology and reduced production of interleukins 1β, IL-5, IL-6, and matrix metalloproteinase 9 (MMP-9) in wild-type asthmatic mice. However, the therapeutic effects of bergenin were significantly attenuated in *Sirt1* ^*fl/fl*^ *-LysMcre* asthmatic mice or following coadministration with the SIRT1 inhibitor EX-527. Further experiments showed that activation of SIRT1 by bergenin deacetylates nuclear factor κB and hinders its nuclear translocation, thereby affecting IL-1β, IL-5, IL-6, and MMP-9 production by regulating transcriptional activity. Our study suggests that bergenin can improve asthma-induced airway inflammation and remodeling by activating SIRT1 in macrophages.

## Introduction

Persistent inflammatory stimulation and airway remodeling are a challenge for asthma treatment (Abe et al., 2021). Activated macrophages are involved in these pathological processes in multiple ways (Liu et al., 2014; Shapouri-Moghaddam et al., 2018; van der Veen et al., 2020), and targeting macrophages may provide new therapeutic strategies for asthma. In our previous study, sirtuin 1 (SIRT1) regulated macrophages and downregulated tumor necrosis factor α (TNF-α) expression through the nuclear factor κB (NF-κB) pathway to suppress airway inflammation (Lai et al., 2021). SIRT1 also reduces airway remodeling *via* epithelial to mesenchymal transition in chronic obstructive pulmonary disease and pulmonary fibrosis (Guan et al., 2020; Li et al., 2021). However, we observed decreased SIRT1 in the lungs of asthmatic mice (Wang et al., 2015). Therefore, drugs that activate SIRT1 may treat asthma.

Bergenin is often used as a SIRT1 agonist (Wang et al., 2017; Pavan Kumar et al., 2019). Our previous study showed that bergenin effectively inhibits TNF-α-induced inflammation of lung epithelial cells (Chen et al., 2020). Moreover, it reduced airway remodeling by inhibiting lung fibroblast activation and extracellular matrix accumulation in a bleomycin-induced mouse model of pulmonary fibrosis (Li et al., 2021). Therefore, we speculated that bergenin has prospects for asthma treatment.

Here, we established a myeloid cell-specific SIRT1 conditional knockout (*Sirt1* ^*fl/fl*^ *-LysMCre*) mouse model of asthma using ovalbumin (OVA) induction and determined the underlying mechanism by which the SIRT1 agonist bergenin acts in the treatment of asthma. Our findings suggest that bergenin activates SIRT1 to regulate production of interleukin 1β (IL-1β), IL-5, IL-6, and matrix metalloproteinase 9 (MMP-9) in macrophages through the NF-κB pathway, thereby having therapeutic utility for improving airway inflammation and remodeling.

## Materials and methods

### Chemicals and reagents

OVA was purchased from Sangon Biotech Co., Ltd. (Shanghai, China; BC Grade). Aluminum hydroxide was purchased from Sigma-Aldrich (St. Louis, MO, United States). Bergenin was purchased from Aladdin Chemistry (Shanghai, China; >98% purity) for cell experiments and Solarbio (Beijing, China; >98% purity) for animal experiments. EX-527, an Ultrasensitive ECL Chemiluminescence Kit, and IL-1β and IL-6 enzyme-linked immunosorbent assay (ELISA) kits were purchased from Beyotime Biotechnology (Shanghai, China). IL-5 ELISA kit was purchased from NeoBioscience Technology Co, Ltd (Shenzhen, China). Antibodies used in western blotting and immunofluorescence are listed in Table 1.

**TABLE 1:** Antibodies used for western blotting, immunohistochemistry, and immunofluorescence.

Antibodies	Brand	Catalog no	Applications
β-actin Mouse Polyclonal	Beyotime		WB: 1:1000
IκB-α Rabbit Polyclonal	Beyotime		WB: 1:1000
SIRT1 Rabbit Polyclonal	Beyotime		WB: 1:1000
Lamin B1 Mouse Polyclonal	Proteintech		WB: 1:1000
NF-κB p65 Rabbit Polyclonal	CST		WB: 1:2000 IF: 1:200
p-NF-κB p65 Rabbit Polyclonal	CST		WB: 1:1000
Ace-NF-κB p65 Rabbit Polyclonal	CST		WB: 1:1000
MMP-9 Rabbit Polyclonal	Abcam		WB: 1:1000

### Animals

Female C57BL/J mice, aged 6–8 weeks, were purchased from Guangdong Medical Laboratory Animal Center (Guangzhou, China). *LysMCre* mice were purchased from The Jackson Laboratories (bar Harbor, ME, United States). *Sirt1* ^*fl/fl*^ mice were purchased from GemPharmatech Co., Ltd. (Nanjing, China). Myeloid cell-specific SIRT1 conditional knockout (*Sirt1* ^*fl/fl*^ *-LysMCre*) mice were obtained by crossing *Sirt1* ^*fl/fl*^ mice with *LysMCre* mice. Female *Sirt1* ^*fl/fl*^ *-LysMCre* mice, age 6–8 weeks, were used for subsequent experiments. Primer sequences for *LysMCre* and *Sirt1* genes are listed in Table 2. All animals were housed under a 12-h light/dark cycle (lights on from 7 am to 7 pm) with controlled room temperature (approximately 25°C) and humidity (55%–70%) in cages (290 × 178 × 160 mm). All experimental procedures were approved by the Animal Ethical Committee of Guangdong Medical University.

**TABLE 2:** Primer sequences used in this study.

Genes	Forward (5′ to 3′)	Reverse (5′ to 3′)
*Sirt1* (m)	CAC​TGT​AAC​TGG​GGG​CAA​CT	CAC​TTC​TTG​TCA​GCG​TCG​AA
IL-6	TAG​TCC​TTC​CTA​CCC​CAA​TTT​CC	TTG​GTC​CTT​AGC​CAC​TCC​TTC
IL-1β	GCA​ACT​GTT​CCT​GAA​CTC​AAC​T	ATC​TTT​TGG​GGT​CCG​TCA​ACT
β-actin	AGT​GTG​ACG​TTG​ACA​TCC​GT	GCA​GCT​CAG​TAA​CAG​TCC​GC
*LysMCre-WT* (m)	TTA​CAG​TCG​GCC​AGG​CTG​AC	
*LysMCre-Mutant* (m)	CCCAG AAATGCCAGATTACG	
*LysMCre-Common* (m)	CTT​GGG​CTG​CCA​GAA​TTT​CTC	
*Sirt1 gene deletion* (m)	oIMR7909: 5′-GGT​TGA​CTT​AGG​TCT​TGT​CTG-3′	
	oIMR7912: 5′-CGT​CCC​TTG​TAA​TGT​TTC​CC-3′	

### Establishment of asthma model and administration of bergenin and EX-527

The asthma model was established as follows: on days 0, 7, and 14, mice were sensitized by an intraperitoneal injection of 200 μL of a phosphate-buffered saline (PBS) suspension containing 50 μg OVA and 2 mg aluminum hydroxide. Next, the sensitized mice were nebulized with PBS containing 5% OVA for 30 min from days 15 to 21. Bergenin was suspended in PBS and administered to mice by gavage at 1 h before nebulized OVA. The SIRT1 agonist EX-527 was dissolved in dimethyl sulfoxide, suspended in 1% CMC-Na, and administered by intraperitoneal injection to mice at 2 h before nebulized OVA. The solution volume for both gavage and intraperitoneal injection was 200 μl. All mice were sacrificed with 1% sodium pentobarbital 24 h after the last nebulized OVA administration, and lung tissue and bronchoalveolar lavage fluid (BALF) were collected.

### Cell culture and viability assay

MH-S cells were maintained in Roswell Park Memorial Institute 1640 medium containing 10% fetal bovine serum at 37°C with 5% CO_2_. The viability of MH-S cells was evaluated using a CCK-8 kit (Beyotime) in accordance with the manufacturer’s instructions. Cells were seeded in 96-well plates and treated with bergenin (0, 10, 20, 40, 60, 80, 100, 200, and 400 μM) for 24 h. The absorbance of each well at 450 nm was determined using a microplate reader.

### qPCR assay

Total RNA was extracted from cells using TRIzol extraction reagent, cDNA was synthesized using PrimeScript RT Master Mix, and target gene expression was detected by qPCR amplification using SYBR^®^ Premix Ex Taq™ II [all reagents were purchased from Takara Biotechnology (Kusatsu, Japan)]. qPCR primer sequences are shown in Table 2 β-Actin was used as an internal reference gene. Relative expression of a target gene was obtained by calculating 2^−ΔΔCt^.

### Enzyme-linked immunosorbent assay

MH-S cells were pretreated with bergenin and/or EX-527 for 1 h, stimulated with TNF-α (20 ng/ml) for 24 h, and then the medium was harvested. Both harvested medium and BALF from mice were centrifuged at 8000 × *g* for 8 min, and supernatants were collected and stored at −80°C for subsequent analysis. IL-1β, IL-5, and IL-6 concentrations in supernatants were measured using ELISA kits in accordance with the manufacturer’s instructions.

### Western blotting

Total proteins were extracted from lungs and cells using radioimmunoprecipitation assay buffer with 1% phenylmethylsulfonyl fluoride. Cytoplasmic and nuclear proteins were isolated with a nuclear-cytoplasmic protein extraction kit. Protein samples were stored at −80°C for subsequent analysis. Samples were separated by 10% sodium dodecyl sulfate polyacrylamide gel electrophoresis and electrotransferred to a polyvinylidene fluoride membrane. The membrane was blocked with 5% dry non-fat milk for 1 h at room temperature and then incubated with a primary antibody at 4°C overnight. Subsequently, the membrane was incubated with a horseradish peroxidase-conjugated secondary antibody for 1 h at room temperature and imaged using an Ultrasensitive ECL Chemiluminescence Kit. Gray values of protein bands were quantified using ImageJ software (Version 1.43; National Institutes of Health, Bethesda, MD, United States).

### Histological staining for lung tissue

Fresh left lung tissues from mice were fixed in 10% formalin for 24 h. Subsequently, the fixed tissue was embedded in paraffin and cut into 5-μm-thick sections. Finally, sections were stained with hematoxylin and eosin, periodic acid-Schiff, or Masson’s trichrome.

### Immunofluorescence

MH-S cells were pretreated with bergenin (60 μM) and/or EX-527 (10 μM) for 1 h and then stimulated with TNF-α (20 ng/ml) for 5 min. After removing the medium and rinsing with PBS, cells were fixed in 4% paraformaldehyde for 15 min, permeabilized in 0.1% Triton X-100 for 10 min, blocked in 5% bovine serum albumin for 30 min, and then incubated with anti-NF-κB p65 antibody or anti-Ace-p65 or anti-SIRT1 antibody overnight at 4°C. Subsequently, cells were incubated with a FITC-conjugated secondary antibody for 1 h at 37°C in the dark. A DAPI-containing anti-fluorescence quencher was applied for 5 min and then the cells were imaged under a confocal microscope (Olympus, Tokyo, Japan).

### Statistical analysis

Statistical analysis was performed using PrismGraph software (Graphpad, San Diego, CA, United States). Data are expressed as the mean ± SEM. Mean differences between two groups were analyzed by t-test. Mean differences among multiple groups were compared by one-way ANOVA and Fisher’s least significant difference test. *p*-values of less than 0.05 were considered significant.

## Results

### Bergenin ameliorates airway inflammation and remodeling in asthmatic mice

**FIGURE 1:**
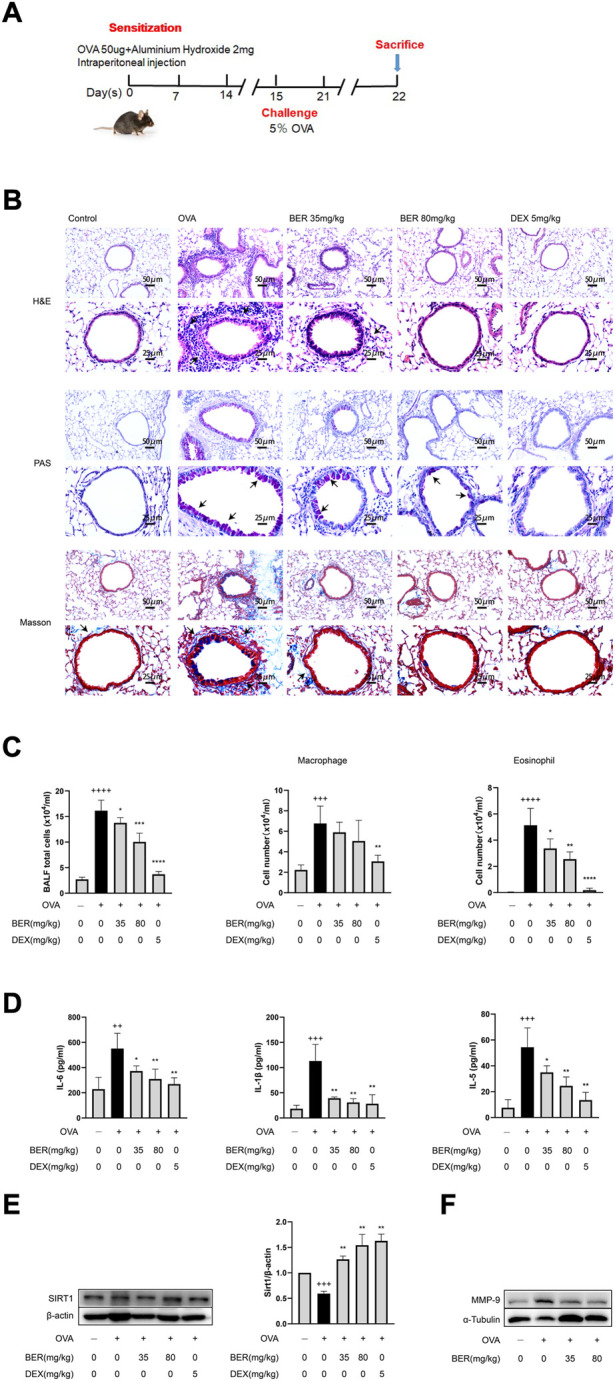
Bergenin ameliorates airway inflammation and remodeling in asthmatic mice. **(A)** Schematic of the allergic asthma model. **(B)** Hematoxylin and eosin (H&E), periodic acid-Schiff (PAS), and Masson staining of lung tissue (scale bar of the upper panel: 50 μm, scale bar of the bottom panel: 25 μm). **(C)** Numbers of total inflammatory cells, macrophages and eosinophils in bronchoalveolar lavage fluid (BALF). **(D)** Interleukin 6 (IL-6), IL-5, and IL-1β protein levels in BALF were measured by ELISAs. **(E)** Western blot analysis of SIRT1 in lung tissue. β-Actin was used as an internal control. **(F)** Western blot analysis of matrix metalloproteinase 9 (MMP-9) in lung tissue. α-Tubulin was used as an internal control. Data are presented as Mean ± SEM of three independent experiments (*n* = 5–8 for each group). ^++^ *p* < 0.01, ^+++^ *p* < 0.001, ^++++^ *p* < 0.0001 vs. control group; *p < 0.05, **p < 0.01, ***p < 0.001, ****p < 0.0001 vs. OVA group.

In the present study, we established a mouse asthma model by OVA induction (Figure 1A) and administered bergenin (35 or 80 mg/kg) or DEX (5 mg/kg) as a positive control by gavage. Bergenin and DEX both significantly reduced inflammatory cell infiltration into lung tissue and inhibited proliferation of goblet cells and collagen fibers (Figure 1B). We also observed significant decreases in total cell numbers and eosinophils in BALF, as well as IL-6, IL-1β, IL-5, MMP-9, and SIRT1 protein expression in asthmatic mice treated with bergenin or DEX (Figures 1C–F). These findings show that bergenin improves airway inflammation and remodeling in asthmatic mice.

### The efficacy of bergenin is attenuated in *sirt1* ^*fl/fl*^ *-lysmcre* asthmatic mice

**FIGURE 2:**
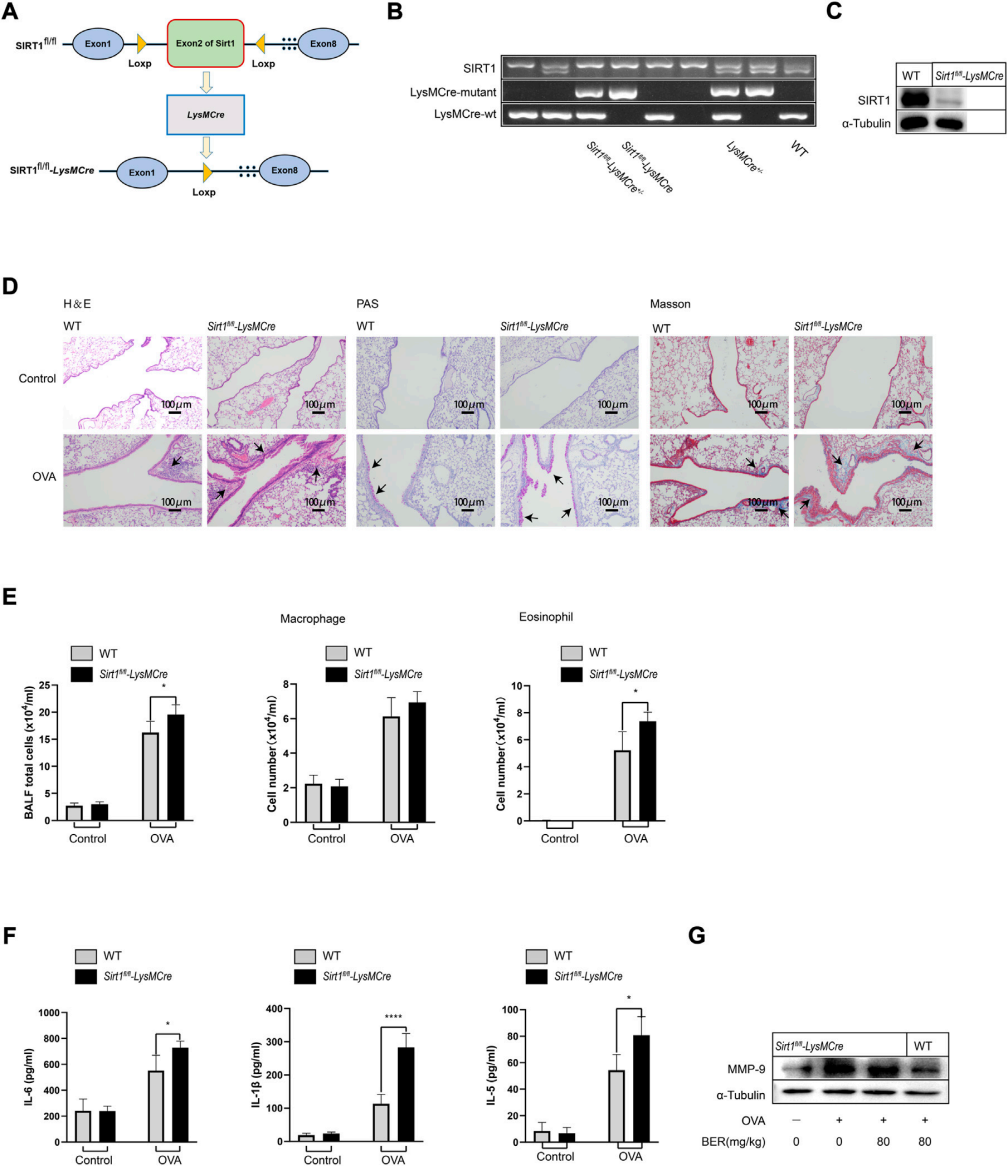
The efficacy of bergenin is attenuated in *Sirt1* ^*fl/fl*^ *-LysMCre* asthmatic mice. **(A)** Schematic of the generation of *Sirt1* ^*fl/fl*^ *-LysMCre* mice. **(B)** Genotyping was performed by PCR using mouse tail genomic DNA. **(C)** Genotyping was performed in BMDMs from *Sirt1* ^*fl/fl*^ and *Sirt1* ^*fl/fl*^ *-LysMCre* mice by western blotting. **(D)** Hematoxylin and eosin (H&E), periodic acid-Schiff (PAS), and Masson staining of lung tissue (scale bar, 100 μm). **(E)** Number of total inflammatory cells, macrophages and eosinophils in bronchoalveolar lavage fluid (BALF). **(F)** Interleukin 6 (IL-6), IL-5, and IL-1β protein levels in BALF were measured by ELISAs. **(G)** Western blot analysis of matrix metalloproteinase 9 (MMP-9) in lung tissue of wild-type (WT) and *Sirt1* ^*fl/fl*^ *-LysMCre* mice. α-Tubulin was used as an internal control. Data are presented as Mean ± SEM of three independent experiments (*n* = 5–8 for each group). *p < 0.05, ****p < 0.0001, vs. OVA-induced *Sirt1* ^*fl/fl*^ *-LysMCre* asthmatic mice.

We generated myeloid-specific SIRT1 conditional knockout (*Sirt1* ^*fl/fl*^ *-LysMCre*) mice and established an asthma model (Figures 2A–C). Staining of lung tissue sections showed that airway inflammation, mucus secretion, and collagen fiber hyperplasia of *Sirt1* ^*fl/fl*^ *-LysMCre* asthmatic mice were significantly aggravated compared with those of wild-type asthmatic mice (Figure 2D). Total cell numbers and eosinophils in BALF and protein expression of IL-1β, IL-6, IL-5, and MMP-9 in lung tissue of *Sirt1* ^*fl/fl*^ *-LysMCre* asthmatic mice were also higher compared with those of wild-type asthmatic mice (Figures 2E–G). These findings highlight the critical role of SIRT1 in asthma.

**FIGURE 3:**
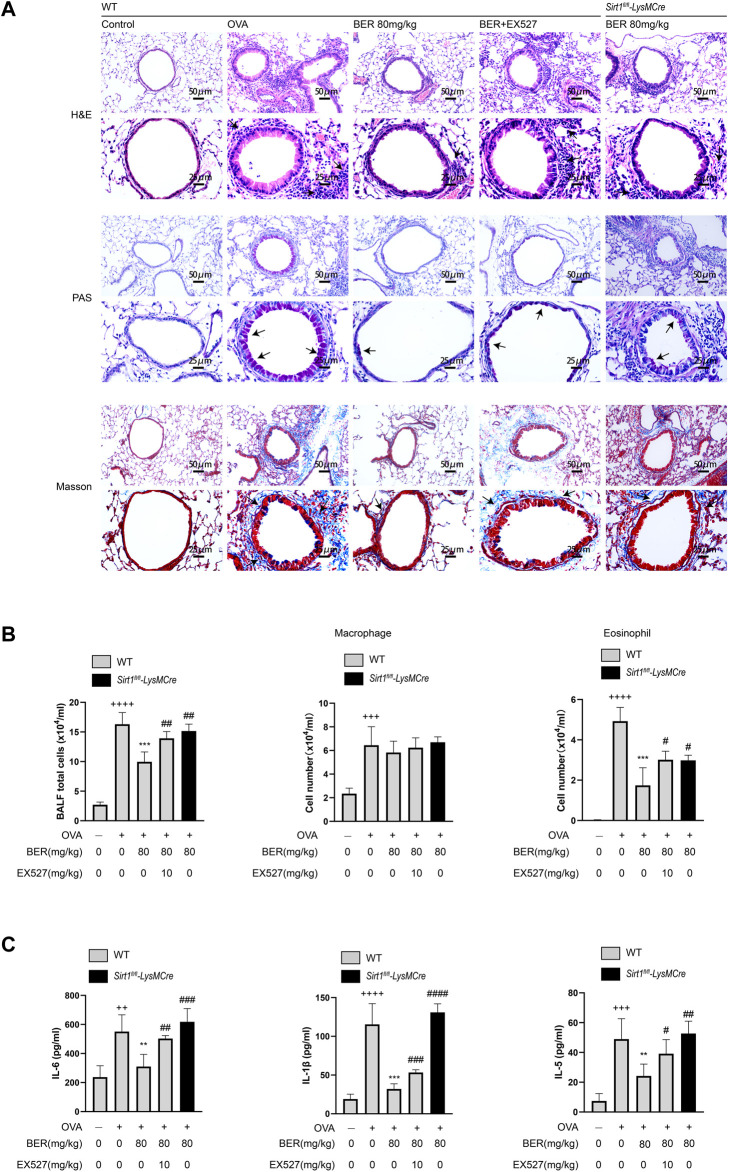
**(A)** Hematoxylin and eosin (H&E), periodic acid-Schiff (PAS), and Masson staining of lung tissue (scale bar of the upper panel: 50 μm, scale bar of the bottom panel: 25 μm). **(B)** Number of total inflammatory cells, macrophages and eosinophils in bronchoalveolar lavage fluid (BALF). **(C)** Interleukin 6 (IL-6), IL-5, and IL-1β protein levels in BALF were measured by ELISAs. Data are presented as Mean ± SEM of three independent experiments (*n* = 5–8 for each group). ^+^^+^ *p* < 0.01, ^+^^+^^+^ *p* < 0.001, ^+^^+^^+^^+^ *p* < 0.0001 vs. control group in WT; **p < 0.01, ***p < 0.001 vs OVA group in WT; ^#^ *p* < 0.05, ^#^^#^ *p* < 0.01. OVA, ovalbumin.

In the experiments described above, we observed that bergenin has potential therapeutic effects such as improving airway inflammation and remodeling in asthmatic mice. However, these effects were not manifested after myeloid knockout of SIRT1. In *Sirt1* ^*fl/fl*^ *-LysMCre* asthmatic mice, we found that inflammatory cell infiltration and hyperplasia of goblet cells and collagen fibers were not (or only partially) alleviated by bergenin (Figure 3A). Similar results were also shown for total cell numbers and eosinophils in BALF and levels of IL-1β, IL-6 and IL-5 (Figures 3B,C). These results suggest that bergenin may play a therapeutic role in asthma by activating SIRT1.

### Bergenin reduces the production of IL-1β, IL-6, and MMP-9 by activating SIRT1 in MH-S cells

**FIGURE 4:**
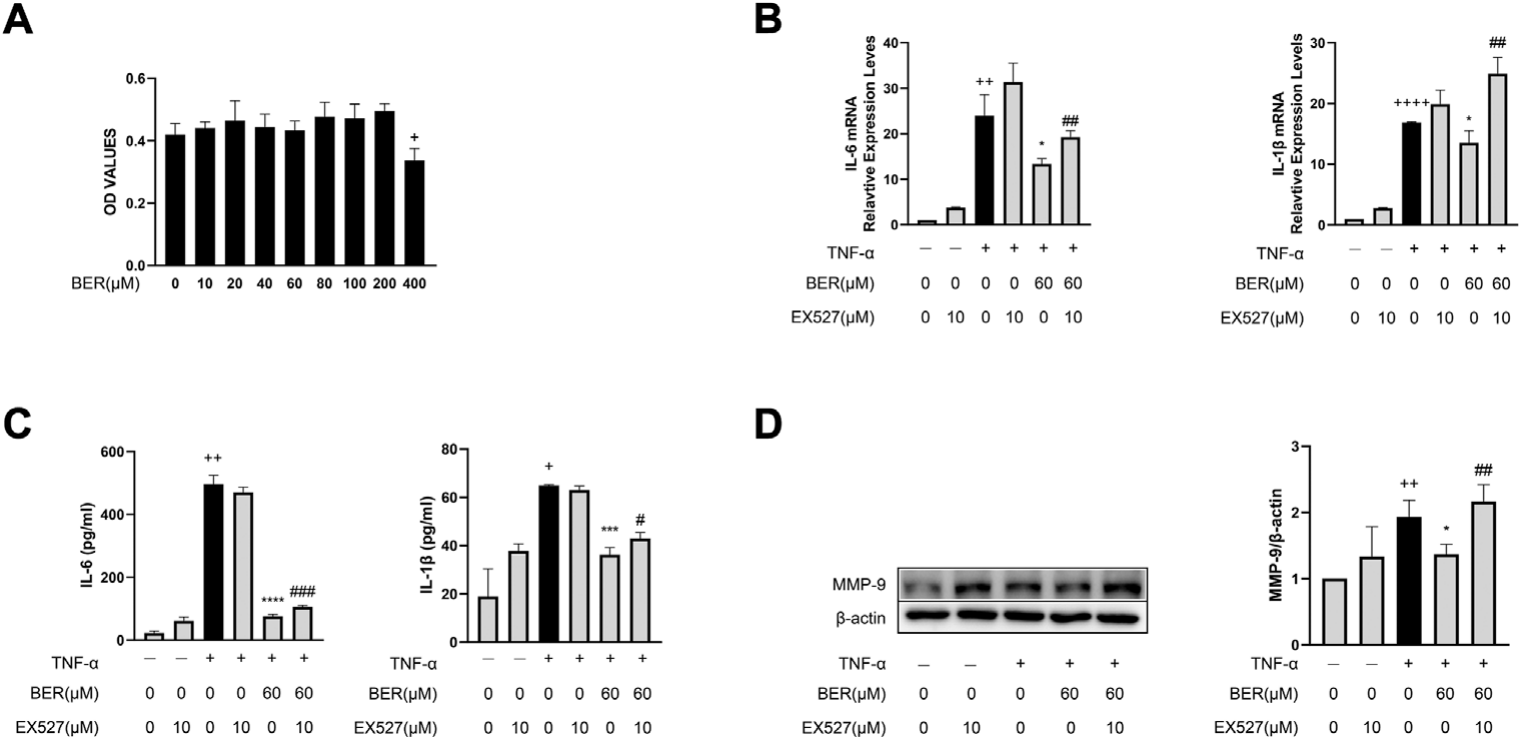
Bergenin reduces production of IL-1β, IL-6, and MMP-9 by activating SIRT1 in MH-S cells. **(A)** Cellular toxicity of bergenin in MH-S cells. **(B)** Interleukin 6 (IL-6) and IL-1β mRNA levels in MH-S cells were analyzed with RT-qPCR. **(C)** ELISA analysis of IL-6 and IL-1β protein concentrations in the culture supernatant of MH-S cells. **(D)** Western blot analysis of matrix metalloproteinase (MMP-9) in MH-S cells. β-Actin was used as an internal control. Data are presented as Mean ± SEM of three independent experiments. ^+^ *p* < 0.05, ^++^ *p* < 0.01, ^++++^ *p* < 0.0001 vs. control in MH-S cells; *p < 0.05, ***p < 0.001, ****p < 0.0001 vs TNF-α alone in MH-S cells, ^#^ *p* < 0.05, ^#^^#^ *p* < 0.01, ^#^^#^^#^ *p* < 0.001 vs TNF-α + Bergenin (60 μM).

To further confirm that bergenin exerts a therapeutic effect by activating SIRT1, we performed *in vitro* experiments using MH-S cells. Bergenin had no significant effect on cell viability at concentrations of 0–200 μM (Figure 4A) and we chose 60 μM for subsequent cell experiments. Our results show that IL-1β, IL-6, and MMP-9 were increased upon TNF-α stimulation, but decreased following pretreatment with bergenin. However, when we inhibited SIRT1 using the SIRT1 inhibitor EX-527, bergenin failed to reduce the production of these inflammatory mediators (Figures 4B–D).

### Bergenin regulates expression of inflammatory mediators in MH-S cells *via* SIRT1-Mediated NF-κB pathway

**FIGURE 5:**
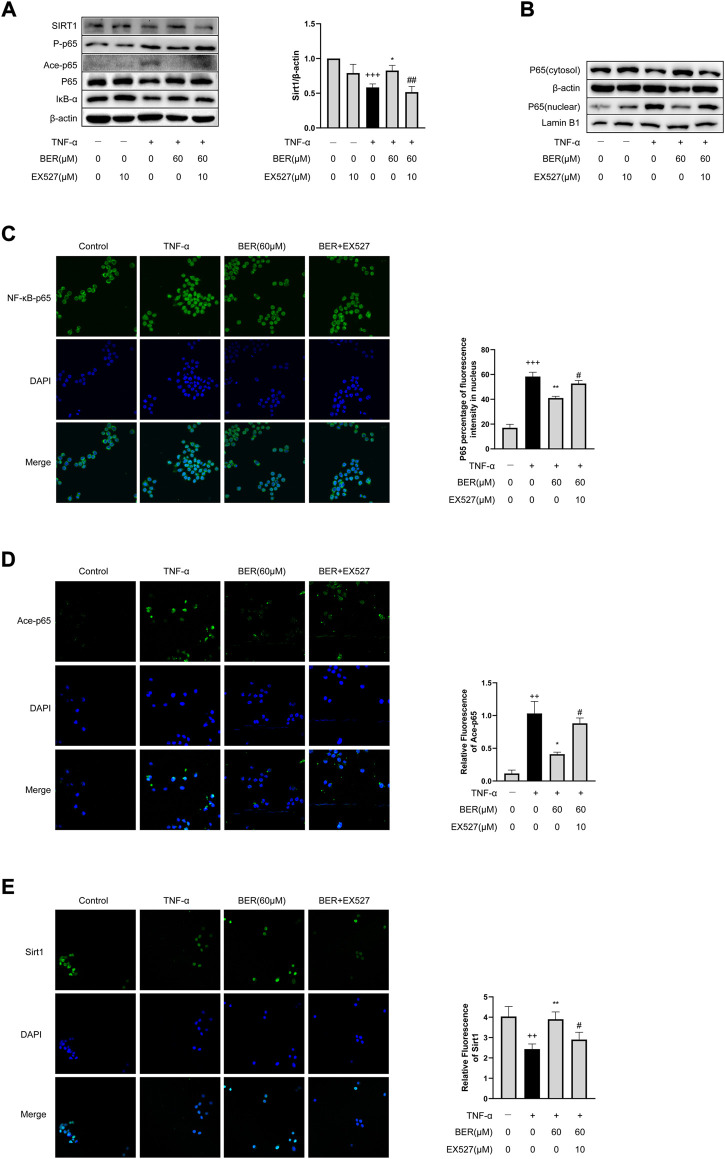
Bergenin regulates expression of inflammatory mediators in MH-S cells *via* the SIRT1-mediated NF-κB pathway. **(A)** Western blot analysis of SIRT1, phospho-NF-κB p65, acetyl-NF-κB p65, NF-κB p65, and IκB-α in MH-S cells. β-Actin was used as an internal control. **(B)** Western blot analysis of NF-κB-p65 nuclear translocation in MH-S cells. β-Actin and lamin B1 were used as internal controls. **(C)** NF-κB p65 nuclear translocation analyzed by immunofluorescence. **(D)** Acetyl-NF-κB p65 nuclear translocation analyzed by immunofluorescence. **(E)** SIRT1 level was determined by immunofluorescence. Data are presented as Mean ± SEM of three independent experiments. ^+^^+^ *p* < 0.01, ^+^^+^^+^ *p* < 0.001 vs control; *p < 0.05, **p < 0.001 vs TNF-α alone; ^#^ *p* < 0.05, ^#^^#^ *p* < 0.01 vs TNF-α + Bergenin (60 μM). NF-κB, nuclear factor κB; TNF-α, tumor necrosis factor α.

To evaluate whether bergenin inhibits IL-1β, IL-6, and MMP-9 expression by regulating the SIRT1-mediated NF-κB pathway, we performed recovery experiments with EX-527 in MH-S cells. Following TNF-α stimulation, we observed downregulation of SIRT1; upregulation of p-p65, and ace-p65; and increased nuclear translocation of p65 in MH-S cells; however, bergenin reversed these changes (Figures 5A,B). When co-administered with EX-527, the inhibitory effects of bergenin on expression of SIRT1, ace-p65 and p-p65, and nuclear translocation of p65 were attenuated (Figures 5A,B,E). Immunofluorescence confirmed that EX-527 attenuated the ability of bergenin to block p65 nuclear translocation, while also inhibiting p65 acetylation (Figures 5C,D).

### Bergenin improves asthmatic airway inflammation and remodeling *via* the SIRT1/NF-κB pathway in a mouse asthma model

**FIGURE 6:**
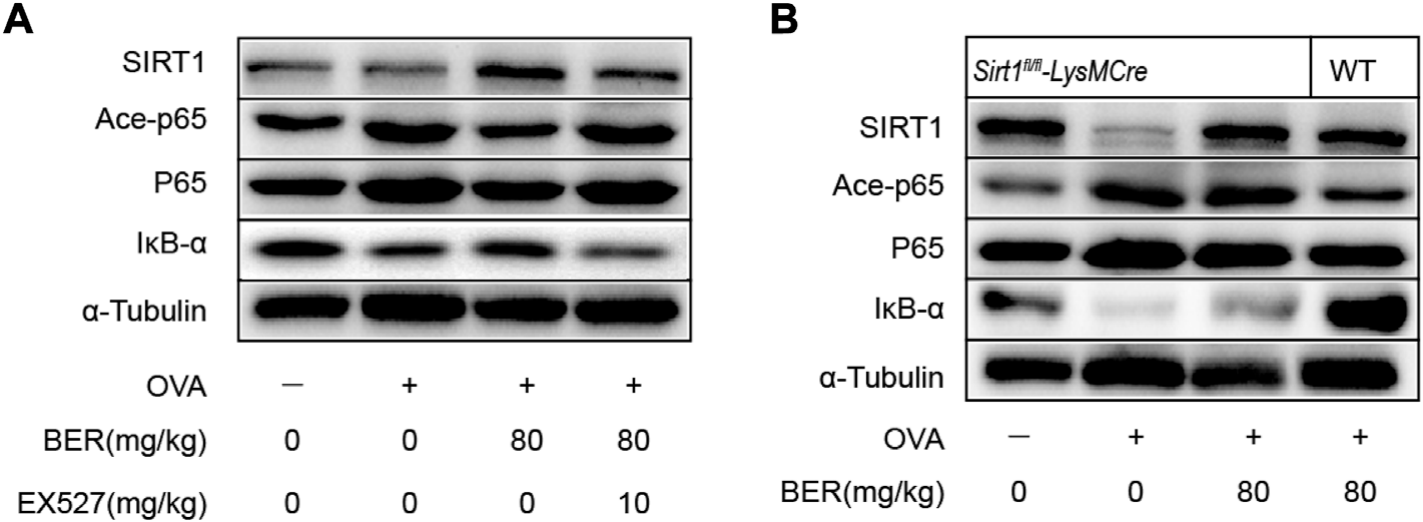
Bergenin improves asthmatic airway inflammation and remodeling *via* the SIRT1/NF-κB pathway in a mouse asthma model. **(A)** Western blot analysis of IκB-α, acetyl-NF-κB p65, and NF-κB p65 in lung tissue. α-Tubulin was used as an internal control. **(B)** Western blot analysis of IκB-α, acetyl-NF-κB p65, and NF-κB p65 in lung tissue of wild-type (WT) and *Sirt1* ^*fl/fl*^ *-LysMCre* mice. α-Tubulin was used as an internal control. NF-κB, nuclear factor κB. (*n* = 5–8 for each group).

Consistent with our *in vitro* results, SIRT1 and IκB-α were downregulated in asthmatic mice, while ace-p65 was upregulated, but bergenin treatment reversed these effects (Figures 6A,B). However, this effect of bergenin was attenuated when co-administered with EX-527 and in SIRT1 myeloid cell-specific knockout asthmatic mice (Figures 3A–C; Figures 6A,B).

## Discussion

In the present study, we observed that bergenin can improve airway inflammation and remodeling in asthmatic mice. In addition, we observed higher expression of SIRT1 in the lung tissue of bergenin-treated asthmatic mice compared with that in the lung tissue of untreated asthmatic mice. Therefore, we speculate that bergenin can potentially contribute to the treatment of asthma by activating SIRT1. To further elucidate the specific mechanism by which bergenin provides treatment of asthma, we used myeloid-specific SIRT1 conditional knockout mice to study the effect of bergenin in asthma models. Our *in vivo* findings demonstrate that myeloid-specific SIRT1 deletion exacerbates airway inflammation and remodeling associated with asthma. Moreover, administration of the SIRT1-activating drug bergenin inhibited these changes in wild-type asthmatic mice, while SIRT1 depletion attenuated the efficacy of bergenin. Furthermore, we confirmed that activation of SIRT1 by bergenin prevented p65 nuclear translocation and inhibited NF-κB transcriptional activity. Collectively, these findings suggest that bergenin has a therapeutic effect on asthma.

Bergenin was used as a SIRT1 agonist for its anti-inflammatory effects observed in our previous study, although another study showed that bergenin also has anti-fibrotic effects in the airway (Chen et al., 2020; Li et al., 2021). In this study, bergenin had a good effect on alleviating OVA-induced asthma in mice. Histochemical staining of lung tissue showed that bergenin significantly reduced inflammatory cell infiltration, numbers of goblet cells, secretion of airway mucus, and deposition of pathological collagen in the airway. Additionally, bergenin inhibited inflammatory mediators IL-6, IL-1β, IL-5, and MMP-9.

Recent findings suggest that macrophages regulate the production of anti-inflammatory cytokines influencing asthma development (Liu et al., 2014; Shapouri-Moghaddam et al., 2018; van der Veen et al., 2020). In our previous study, we established a *Sirt1* ^*fl/fl*^ *-LysMCre* asthmatic mouse model to elucidate the underlying mechanism by which SIRT1 regulates macrophages through the ERK/p38 MAPK pathway to suppress airway inflammation and mucus production (Lai et al., 2021). In this study, we found that *Sirt1* ^*fl/fl*^ *-LysMCre* asthmatic mice exhibited more severe airway inflammation and remodeling than wild-type mice, and expression of IL-1β, IL-5, IL-6, and MMP-9 was also significantly increased. However, bergenin treatment inhibited these changes in wild-type asthmatic mice, while SIRT1 depletion attenuated the efficacy of bergenin and weakened these beneficial changes. These results suggest that bergenin elicits a therapeutic effect in asthmatic mice by activating SIRT1.

IL-1β, IL-6, and MMP-9, which are regulated by the NF-κB pathway, are major mediators of airway inflammation and remodeling in asthma (Eberhardt et al., 2000; Belleguic et al., 2002; Fu et al., 2015; Peters et al., 2016; Chen et al., 2020). When the NF-κB complex is activated, IκB-α dissociates from the complex and subsequent phosphorylation and acetylation of p65 promote nuclear translocation of NF-κB, thereby enhancing its transcriptional activity and promoting the expression of inflammatory mediators. These findings are consistent with previous studies (Chung et al., 2010; Wang et al., 2017). To fully elucidate the role of SIRT1 in this process, we performed *in vitro* and *in vivo* experiments with myeloid-specific cell knockout of SIRT1, the SIRT1 inhibitor EX-527, and bergenin. Our results show that airway inflammation and remodeling were improved by bergenin treatment. However, when SIRT1 was knocked down or inhibited, its reduced deacetylation ability was unable to regulate NF-κB transcriptional activity, decreasing the therapeutic effect. Although NF-κB transcriptional activity also involves phosphorylation of p65, our results demonstrate an important role of SIRT1 deacetylation in regulating NF-κB transcriptional activity.

**FIGURE 7:**
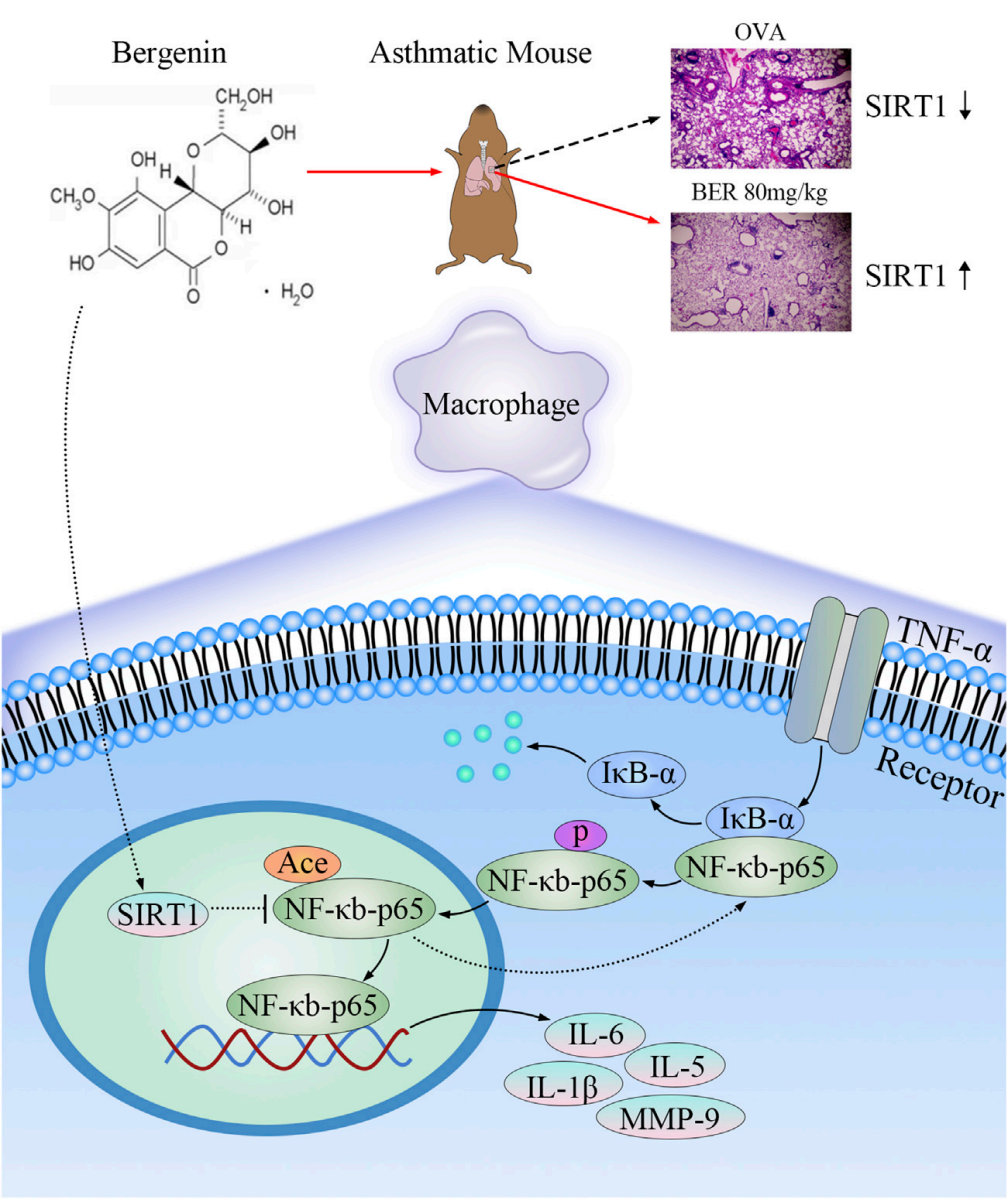
Mechanisms underlying the anti-inflammatory and anti-airway remodeling effects of activated SIRT1. Bergenin activates SIRT1 to deacetylate nuclear factor κB (NF-κB) p65 and inhibit expression of interleukin 6 (IL-6), IL-5, IL-1β, and matrix metalloproteinase 9 (MMP-9), thereby improving airway inflammation and remodeling in asthma.

In conclusion, our study shows that bergenin regulates the NF-κB pathway and blocks p65 translocation into the nucleus by activating SIRT1, thereby reducing the release of proinflammatory factors (such as IL-1β, IL-5, IL-6, and MMP-9 by macrophages) to play a role in asthma treatment (Figure 7).

## Data Availability

The original contributions presented in the study are included in the article/supplementary material, further inquiries can be directed to the corresponding authors.
